# Developmental toxicity of pharmaceuticals in lower vertebrates

**DOI:** 10.1186/1751-0147-54-S1-S13

**Published:** 2012-02-24

**Authors:** Cecilia Berg, Björn Brunström, Ingvar Brandt

**Affiliations:** 1 Dept of Environmental Toxicology, Uppsala University, Sweden

## 

The release of human pharmaceuticals in the aquatic environment has become an issue of societal concern. More than 160 Active Pharmaceutical Ingredients (APIs) have been detected in surface waters, although generally at low concentrations (ng-ug/l). Most available ecotoxicological information on these pharmaceuticals originates from acute testing using algae, Daphnia and fish; results from long-term exposures are still very scarce, particularly in vertebrates. There is, however, emerging evidence to suggest that several human and veterinary pharmaceuticals may pose a serious threat to aquatic wildlife. Still, there are only two examples where exposure to pharmaceuticals has been indisputably linked to adverse effects in wild vertebrates, i.e. the feminizing effects of the synthetic estrogen ethinylestradiol (EE_2_) in fish and the decline in vulture populations caused by the non-steroidal anti-inflammatory drug diclofenac in India and Pakistan.

We are currently studying effects of steroidal hormone APIs on sex organ development following exposure during early life-stages in birds (egg injection), frogs and fish (exposure via ambient water). The results show that the developing Müllerian duct (absent in fish) is a highly sensitive target for endocrine disruption and developmental toxicity in amphibians and birds.

In birds, both diethylstilbestrol (DES) and EE_2_, injected into the embryonated egg, cause a disrupted differentiation and malformations in different parts of the oviduct, including the shell gland. Egg injection of EE_2_ also resulted in eggshell thinning in the next generation of eggs, and a dramatically altered pattern of expression of the enzyme carbonic anhydrase in the shell gland. Eggshell thinning is probably the most serious ecotoxic effect that has afflicted avian wildlife. Based on these and other data, we propose that eggshell thinning could represent a developmental effect in the Müllerian duct rather than a direct effect of pollutants in the oviduct of the adult egg-laying bird.

The Müllerian duct also proved to be a sensitive target of toxicity in frogs. Exposure of tadpoles to EE_2_ resulted in a skewed sex ratio with almost complete feminization at an ecologically relevant concentration. A partial or complete lack of oviducts was observed in these frogs, resulting in ovulation directly into the abdominal cavity. Also the gestogenic pharmaceutical levonorgestrel (LNG), used in contraceptives and other hormone therapies, has recently been demonstrated to give rise to oviductal agenesis following developmental exposure. Unlike the impact in EE_2_-exposed frogs, the adult ovaries of the LNG-exposed frogs also displayed an increased fraction of immature oocytes, arrested in early meiotic prophase. We conclude that both EE_2_ and LNG are potent developmental toxicants at environmentally relevant exposures in frogs, giving rise to dysfunctional female sex organs with subsequent sterility.

In conclusion, exposure to pharmaceutical steroids during early life stages can permanently impair female reproductive organ development and fertility in lower vertebrates.

